# Computer-Assisted Image Processing System for Early Assessment of Lung Nodule Malignancy

**DOI:** 10.3390/cancers14051117

**Published:** 2022-02-22

**Authors:** Ahmed Shaffie, Ahmed Soliman, Amr Eledkawy, Victor van Berkel, Ayman El-Baz

**Affiliations:** 1 BioImaging Laboratory, Department of Bioengineering, University of Louisville, Louisville, KY 40292, USA; amshaf02@louisville.edu (A.S.); asnaee01@louisville.edu (A.S.); 2 Computer Science Department, Faculty of Computers and Information, Mansoura University, Mansoura 35516, Egypt; amr.eledkawy@mans.edu.eg; 3 Department of Cardiovascular and Thoracic Surgery, University of Louisville, Louisville, KY 40202, USA; victor.vanberkel@louisville.edu

**Keywords:** lung cancer, HOG, LBP, MGRF, CSS, spherical harmonics, autoencoder, CT image

## Abstract

**Simple Summary:**

Lung cancer is the second most common cancer in men after prostate cancer and in women after breast cancer, but it is the leading cause of cancer death among both genders. This manuscript proposes a new computer-aided diagnosis system that uses only a single computed tomography scan to diagnose the pulmonary nodule as benign or malignant. This system helps in the early detection of the pulmonary nodules and shows its ability to identify the pulmonary nodules precisely.

**Abstract:**

Lung cancer is one of the most dreadful cancers, and its detection in the early stage is very important and challenging. This manuscript proposes a new computer-aided diagnosis system for lung cancer diagnosis from chest computed tomography scans. The proposed system extracts two different kinds of features, namely, appearance features and shape features. For the appearance features, a Histogram of oriented gradients, a Multi-view analytical Local Binary Pattern, and a Markov Gibbs Random Field are developed to give a good description of the lung nodule texture, which is one of the main distinguishing characteristics between benign and malignant nodules. For the shape features, Multi-view Peripheral Sum Curvature Scale Space, Spherical Harmonics Expansion, and a group of some fundamental morphological features are implemented to describe the outer contour complexity of the nodules, which is main factor in lung nodule diagnosis. Each feature is fed into a stacked auto-encoder followed by a soft-max classifier to generate the initial malignancy probability. Finally, all these probabilities are combined together and fed to the last network to give the final diagnosis. The system is validated using 727 nodules which are subset from the Lung Image Database Consortium (LIDC) dataset. The system shows very high performance measures and achieves 92.55%, 91.70%, and 93.40% for the accuracy, sensitivity, and specificity, respectively. This high performance shows the ability of the system to distinguish between the malignant and benign nodules precisely.

## 1. Introduction

According to the World Health Organization (WHO), cancer is the second leading cause of death globally after heart disease. Since cancer has one of the greatest economic impacts on society and people, cancer prevention and control is now a top priority in medicine [1]. The fight against cancer is based on three main pillars that comprise the development and application of (1) disease prevention measures, (2) new methods for disease screening and early detection, and (3) new cancer treatment drugs and therapies. Compared to all type of cancers, lung cancer is the leading cause of all cancer-related deaths [2]. The typical symptoms include weight loss, sore throat, coughing, exhaustion, chest inflammation, chest pain, and hemoptysis [3]. In 2020, 2,206,771 cases were diagnosed with lung cancer worldwide, and 1,796,144 deaths from lung cancer were recorded, accounting for approximately 18% of total mortality in many countries [4]. Lung cancer is the most prevalent cancer in men and the second most prevalent cancer in women after breast cancer [5,6]. Unfortunately, the majority of patients arrive with late-stage disease (stages III and IV), and there are few viable treatment choices, resulting in significant fatality rates [7]. Another significant factor decreasing the mortality rate of this condition is the difficulty of lung cancer diagnosis, where extreme delays in recognizing cases of lung cancer may cause the deterioration of the patient’s condition. This explains the need for diagnostic and clinical protocols to be successful and incorporated promptly [8]. Early detection of lung cancer enhances treatment effectiveness and boosts the five-year survival rate from 17.7% to 55.2% [9].

Modalities that are used for lung cancer screening inlcude: computer-assisted tomography (CT), chest X-ray, sputum cytology, fluorescence bronchoscopy, positron emission tomography (PET), and magnetic resonance imaging (MRI), with CT being the most common used modality [10,11,12,13,14]. CT scans help to diagnose lung cancer more precisely and thereby reduce mortality by 20% [15]. The problem with CT scans in the detection of lung cancer is that they present a challenging task for doctors, since the amount of data saved in the CT screening takes a long time to examine. By using automated nodule diagnostic systems, we can save time and costs and avoid the risks of invasive surgical procedures, and this has been proven in [16], where breast cancer, melanoma, cervical cancer, and colorectal cancer death rates were shown to have fallen significantly as a result of early cancer detection with effective screening tests. Many academics are working on new frameworks for computer-assisted diagnostic (CADx) systems to aid clinicians in analyzing the massive amount of information, using machine-learning and image-processing techniques to aid in the diagnosis of lung cancer in a timely and accurate manner [17,18,19,20]. Lakshmanaprabu et al. [21] classified lung CT scans using Linear Discriminate Analysis (LDA), Optimal Deep Neural Network (ODNN), and Modified Gravitational Search Algorithm (MGSA). The extracted set of features were then inputted into the ODNN classifier and optimized using MGSA. They tested their method using 50 low-dosage lung cancer CT images from the Early Lung Cancer Action Program (ELCAP) Public Lung Image Database. Their approach achieved 96.2% sensitivity, 94.2% specificity, and 94.56% accuracy. Bhatia et al. [22] pre-processed CT scans to extract Regions of Interest (ROI) that may be vulnerable to cancer. The features are extracted from the ROI using UNet and ResNet models. Their proposed system is tested on the Lung Image Database Consortium and Image Database Resource Initiative (LIDC-IRDI) dataset using 10-fold cross-validation. The highest accuracy that has been reached is 84% which is achieved using an ensemble of Random Forest and XGBoost classifier. Bhandary et al. [23] proposed a Modified AlexNet (MAN) deep-learning framework to detect lung pneumonia and cancer. The lung CT scan is first pre-processed using a threshold filter, and then the features are extracted using the Ensemble-Feature-Technique by integrating the deep and handcrafted features. The proposed framework is tested on the LIDC-IDRI dataset and achieved 97.27% classification accuracy. Asuntha and Srinivasan [24] pre-processed the CT scan using histogram equalization and an Adaptive Bilateral Filter. The Artificial Bee Colony segmentation approach is then applied to the enhanced CT scan to extract lung regions. They then extract textual, geometric, volumetric, and intensity features from the enhanced image using techniques such as the Histogram of oriented Gradients (HoG), wavelet transform-based features, Local Binary Pattern (LBP), and Zernike Moment. The selected features are then fed into a Convolutional Neural Network (CNN). The proposed approach is tested on the LIDC-IDRI benchmark dataset and real-time dataset. The proposed framework achieved better results than standard techniques. Another CAD system was proposed by Shakeel et al. [25]. They pre-processed the CT image using the weighted mean histogram equalization approach. The image was then fed into an improved profuse clustering technique to segment the ROI from the image. Spectral features were extracted from the ROI, including mean, third-moment skewness, standard deviation, and fourth-moment kurtosis. Extracted features were then fed into a deep-learning instantaneously trained neural network. The proposed framework was tested on lung CT images collected from the Cancer Imaging Archive (CIA) dataset. The framework achieved 98.42% accuracy. Togaçar et al. [26] used the AlexNet model to extract features from the CT image, where the features are obtained from the last fully-connected layer of the model. The selected features were then fed into linear regression (LR), LDA, decision tree (DT), SVM, k-nearest neighbor (kNN), and softmax classifiers. The proposed framework was tested on a dataset collected from 69 different patients from the CIA. The best test results were 99.51% accuracy, 99.32% sensitivity, and 99.71% specificity. The best results were obtained through AlexNet, mRMR, and the kNN pipeline [27]. Prabukumar et al. [28] segmented the CT image to extract ROI using Fuzzy C-Means (FCM) and region-growing segmentation techniques. The geometric, textural, statistical, and intensity features were then extracted from the segmented ROI. The framework used the SVM classifier and was tested on the ELCAP public lung database and achieved 98.51% accuracy, 98.13% sensitivity, and 98.79% specificity. Kavitha et al. [29] proposed an Efficient Classification Model for Cancer Stage Diagnosis (ECM-CSD) using FCM and SVM techniques based on input lung CT scans. First, the input CT image is pre-processed using Gaussian and Gabor filters. Features are extracted from the enhanced image based on a Gray-Level Co-occurrence Matrix (GLCM) and is then fed into the FCM to segment the lung cancer ROI. The ROI features are then fed into the SVM classifier to classify the stage of the cancer. The proposed model was tested on the LIDC-IDRI benchmark dataset and achieved 93% accuracy. Zhang et al. [30] used transfer learning to tackle the problem of insufficient samples for lung nodule classification. The lung CT scan is first pre-processed and then given to the LeNet-5 model that is used to classify the CT image as benign or malignant, then classified into different levels of malignancies (serious or mild). The transfer learning model is validated on the LIDC-IDRI dataset. The researchers augmented the training data of serious-malignant- and mild-malignant-classified images by performing shifts and rotations on each nodule’s data. They used a 10-fold cross-validation testing strategy and achieved 97% accuracy for classifying benign and malignant nodules and achieved 96.7% accuracy for classifying malignancy levels. Alakwaa et al. [31] proposed a lung cancer diagnosis method based on lung CT scans acquired from the LUNA16 challenge. The image is first thresholded to segment out lung tissue from the rest of the CT scan. Marker-driven watershed segmentation is then used to filter noise and include voxels from the edges. To find nodule candidates in the CT scans, a modified U-Net trained on LUNA16 data was employed. Only candidate nodules in the segmented lungs were fed into 3D CNNs to classify the CT scan as positive or negative for lung cancer. The accuracy of the test set was 86.6%.

The present diagnostic procedures have the following flaws: (i) some systems ignore any spatial interaction by using HU values as an appearance feature; (ii) some systems are inaccurate due to their sensitivity to segmentation due to the use of simple shape features; (iii) some systems depends on black boxes of Neural Networks, which are very hard to interpret. To avoid all these problems, two groups of features that describe the nodule texture and shape have been developed. The texture appearance features group involves not only the HU value of the voxel, but also its relationship with the surrounding neighbors. The shape features group combines complex and simple shape descriptors in order not to be very sensitive for the segmentation process and capture the general contours look.

## 2. Materials and Methods

**Figure 1:**
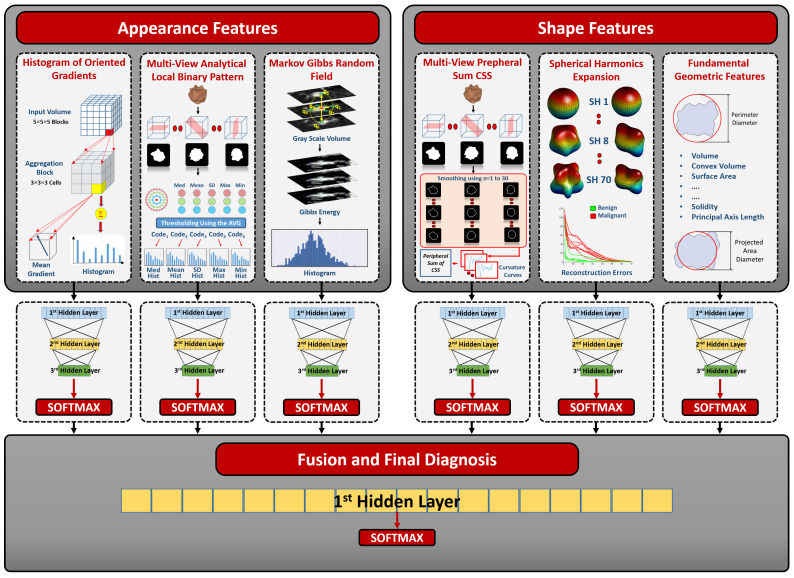
Our framework for pulmonary nodules classification.

The proposed noninvasive lung cancer detection framework combines the texture and shape characteristics of the pulmonary nodule to distinguish between malignant and benign nodules. All these characteristics are extracted from a once-computed tomography scan. Figure 1 illustrates the general idea of the proposed framework and all its details are listed in the following subsections.

### 2.1. Appearance Features

The pulmonary nodule appearance in the CT images, which is also known as nodule texture, is considered a powerful differentiation characteristic for nodule malignancy. This texture is correlated with the nodule growth rate, as high growth rate leads to a non-uniform texture for the nodule appearance, which results in variations in the Hounsfield unit (HU) values in the CT images.

This variation in the texture appearance between malignant and benign nodules is modeled using three different features, namely, MGRF, Multi-view ALBP, and 3D HOG, which are described in the following sections.

#### 2.1.1. Multi-View Analytical Local Binary Pattern

Cancerous pulmonary nodules grow rapidly compared to benign ones, which results in their texture being irregular. These texture differences lead to a distinction in the Hounsfield unit values and will be used to distinguish between the malignant and benign nodules. A Multi-view ALBP descriptor is developed to accurately model the nodule texture and to add the spatial datum of the voxel’s neighbors. This descriptor is an improvement from the traditional LBP and is used to reduce the noise effect on the CT scans and to give a precise model for the nodule texture. This section will start with a brief description of the traditional LBP followed by a presentation of our proposed Multi-view ALBP.

##### The Traditional Local Binary Pattern (LBP)

LBP is a well-recognized feature for texture classification and was introduced by Ojala [32,33]. In the traditional LBP, the local neighborhood around each pixel is thresholded at the central pixel value and the output binary string is considered as a binary number that is weighted as follows:

(1) $$LBP_{N,R}=\sum_{i=1}^{N}2^{i-1}*f\left(g_{i}-c\right),$$

where *c* denotes the gray level of the central pixel, $g_{i}$ denotes the gray level of *N* neighbors that are evenly distributed on a circle of radius *R* around the central pixel, and finally Equation (2) shows the definition of f(x).

(2) $$f\left(x\right)=\left\{\begin{array}{cc} 1 & \mathrm{if}\;x\geq 0\\ 0 & otherwise \end{array}\right..$$

Assuming that the central pixel coordinate is the origin, (0,0), then the coordinates of its *N* neighbors on a circle of radius *R* can be calculated by (−Rsin(2πi/N),Rcos(2πi/N)). In the case where the coordinates are not located perfectly on the grid to obtain the gray level, it will be estimated by resampling using bi-linear interpolation. The LBP could be applied in the three dimensions by considering all the neighbors on a sphere of radius *R*, which will lead to a mass of calculations and a huge running time without an effective accuracy enhancement. The information inherited in the 3D volume could be utilized by extracting various groups of 2D cross-sections for the 3D volumes in fixed planes.

Sometimes, undesirable noise or deformation happens to the CT scans, due to different reasons, such as the number of photons used in the examination, the slice thickness, and the patient size. Using a hard threshold in the traditional LBP makes it very sensitive to the noise, which makes it not suitable for the nodule diagnosis application. Here are some problems that may happen when the traditional LBP is used:

(i) When the nodule has a uniform texture (as in the case of benign nodules), all the neighboring pixels should have the same label, either 1 or 0. However, if a tiny noise is added to the scan (while using a hard threshold), the descriptor will label some pixels 1 and some pixels 0, depending on the noise location.

(ii) When all the neighbor pixels are greater than the pixel in the center, the traditional LBP will label all of them 1 without differentiating between the pixels that have a large difference and those that have a small one. In a similar way, when all the neighboring pixels are less than the pixel in the center, the traditional LBP will label all of them 0 without differentiating between the pixels that have large difference and small one.

##### Analytical Local Binary Pattern

**Figure 2:**
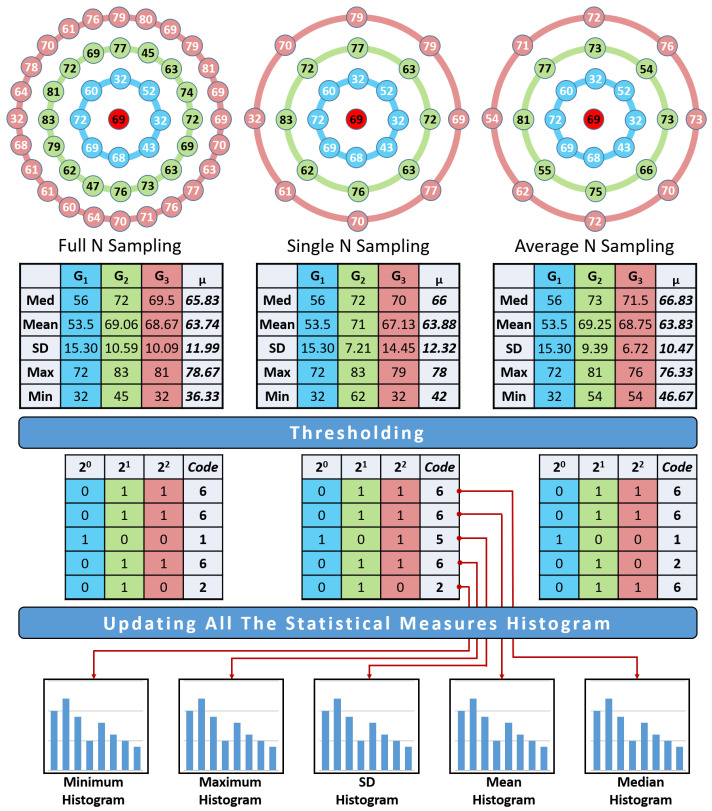
Illustration of ALBP code Calculation.

In order to overcome the mentioned problems, instead of comparing the central pixel to its surrounding neighbors, we started to compute some information about the distribution of the surrounding pixels’ gray levels and compare it to the averages of these values, such as: mean, standard deviation, median, maximum, and minimum. The calculations for the feature vector could be divided into the following three phases (see Figure 2).

(i) Surrounding pixel resampling.

**Figure 3:**
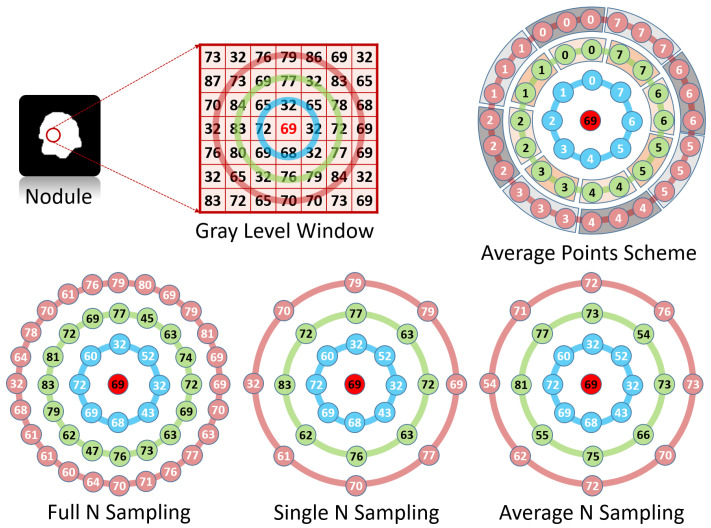
Different examples of the resampling techniques.

To obtain more information about the surrounding pixels, different levels of neighborhood are involved, using different values for the radius *R*. For each level *R*, there is a vector $G_{R}$ that contains all the central pixel neighbors that are evenly distributed on the circle with the specified radius. When the level *R* increases, more pixels are expected to become involved in the calculation. There are three sampling methodologies that are used to resample the surrounding pixels, namely; full *N*, single *N*, and average *N* (see Figure 3).

The full *N* resampling technique tries to involve all the points on the circle around the center. Of course, it is impossible to add all the points on the circle, as it is an infinite number. The number of points that will be used on each level will increase with respect to the level number. In the full N resampling, the number of points at any level *R* will be 8∗R. In other words, every time the level increases by one, 8 more points are added. The drawback of that technique is the huge amount of computations, especially when the *R* is large, and there are points that need bi-linear interpolation. To overcome this issue, the single *N* resampling technique can be utilized, as it will only involve a fixed number of pixels (8 pixels on each level regardless of the *R*).This technique will solve the problem of the computation cost but will lose a lot of data, especially for large values of *R*. To overcome this issue and to compromise between the previous two techniques, an average *N* resampling method could be used. Average *N* resampling uses all the pixels and calculates the average of 8 sets of the pixels. This technique allows us to use the the full amount of available data and reduce them to the same number of the single *N* resampling. All these resampling techniques are tested and compared to obtain the highest classification accuracy.

(ii) Gray-level distribution analysis.

Adding more than one level of pixels will increase dimensionality. So, there is a real need to reduce the dimensionality without losing any texture information. The statistical analysis of the gray level distribution will reduce the dimensionality after taking into account the whole information and present it in a way that makes it easy to analyze. The median value that separates the largest half from the smallest half is one of the used measures. The main disadvantage of that measure is not taking into account the whole dataset (the median value will remain the same regardless of the increase in the highest value or the decrease in the lowest value). So, we need to add more measures such as the mean, which indicates the data average, and the standard deviation, which shows how much the data differs from the average. The maximum and minimum have also been added to the list of the statistical measures that are calculated for the gray level of the neighborhood pixels. All these measures will give summarized information about the gray-level distribution that will enable us to distinguish between the homogeneous texture and the nonhomogeneous texture that characterize the benign and malignant nodules, respectively. For every level *R*, a set of the mentioned statistical measures are calculated for the vector $G_{R}$. This representation reduces the dimensionality from any domain size to only a five-real-numbers vector, that represents the five statistical measures. The expected output from this phase is a vector of size five for each level of radius *R*.

(iii) Construction of the feature vector.

As mentioned above, the traditional LBP and most of its variations use the central pixel to threshold the surrounding neighbor pixels. The surrounding pixels on the gray level are replaced by the distribution analysis measures vector which is calculated from the previous phase. This vector is thresholded using the average of every statistical measure over all the levels. This thresholding vector is denoted by μ and could be calculated using the following equation:

(3) $$\mu =\frac{1}{R}\left[\begin{array}{c} \sum_{i=1}^{R}Median_{i}\\ \sum_{i=1}^{R}Mean_{i}\\ \sum_{i=1}^{R}SD_{i}\\ \sum_{i=1}^{R}Min_{i}\\ \sum_{i=1}^{R}Max_{i} \end{array}\right].$$

The binary code signature will be calculated for every statistical measure separately and its histogram will be updated separately. For example, the binary code for the mean could be calculated using the following equation:

(4) $$ALBP_{R}=\sum_{i=1}^{R}2^{i-1}*f\left(mean\left(G_{i}\right)-\mu \left(mean\right)\right),$$

where:

(5) $$f\left(x\right)=\left\{\begin{array}{cc} 1 & \mathrm{if}\;x\geq 0\\ 0 & otherwise \end{array}\right..$$

#### 2.1.2. A 3D Histogram of Oriented Gradients

**Figure 4:**
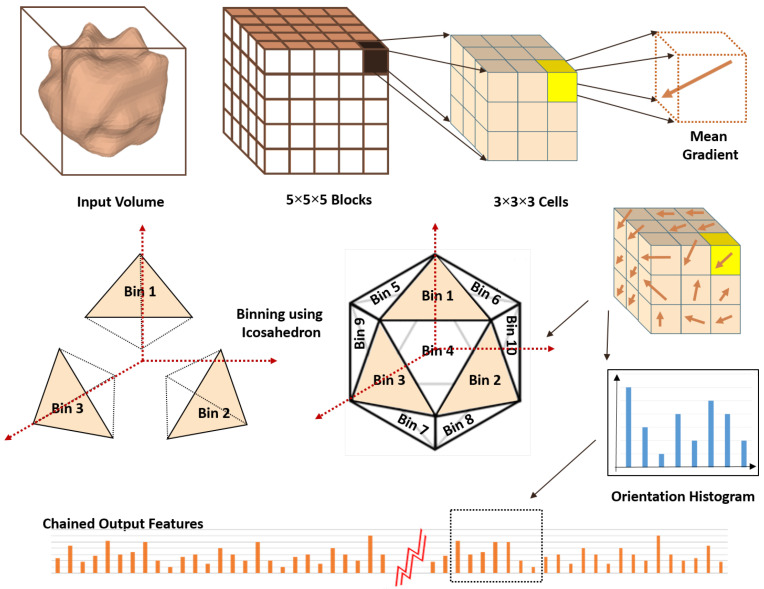
Illustration of histogram of oriented gradient calculation steps.

The HOG is a well-known descriptor that is used in imaging analysis, especially in object detection [34]. The massive calculations of this feature make it time consuming, and this was the main motivation to find a fast and accurate way to implement it, as will be discussed later in this section. The main reason for using the HOG is that it involves the connection between the voxels values in addition to their locations. Figure 4 illustrates the proposed steps to build the HOG feature vector.

##### Calculation of the Mean Gradient

Gradient computation contains massive repeated calculations as it deals with 3D volume. In order to avoid this huge amount of calculations, an intermediate volume, called integral volume, is proposed, which is inspired from the integral image [35]. Considering one of the volume corners as an origin (0,0,0), the value of any voxel in the integral gradient volume can be calculated as the summation of all the partial derivatives of all the voxels that lay between these points and the origin in all axes directions. The integral volume values can be computed utilizing the following equations:

(6) $$\bar{intVol}=\left({intVol}_{x},{intVol}_{y},{intVol}_{z}\right),$$

(7) $${intVol}_{x}=\sum_{\overset{´}{x}\leq x,\overset{´}{y}\leq y,\overset{´}{z}\leq z}V_{dx}\left(\overset{´}{x},\overset{´}{y},\overset{´}{z}\right),$$

(8) $${intVol}_{y}=\sum_{\overset{´}{x}\leq x,\overset{´}{y}\leq y,\overset{´}{z}\leq z}V_{dy}\left(\overset{´}{x},\overset{´}{y},\overset{´}{z}\right),$$

(9) $${intVol}_{z}=\sum_{\overset{´}{x}\leq x,\overset{´}{y}\leq y,\overset{´}{z}\leq z}V_{dz}\left(\overset{´}{x},\overset{´}{y},\overset{´}{z}\right),$$

where $V_{dx}$,$V_{dy}$,$V_{dz}$ are the partial derivatives with respect to x,y,z, respectively. The entire volume can be computed in order of $L^{3}$, where *L* is the volume–gradient side length utilizing the following equations in one pass.

(10) $$line\left(x,y,z\right)=line\left(x,y-1,z\right)+V_{dx}\left(x,y,z\right),$$

(11) plane(x,y,z)=plane(x−1,y,z)+line(x,y,z),

(12) $${intVol}_{x}\left(x,y,z\right)={intVol}_{x}\left(x,y,z-1\right)+plane\left(x,y,z\right),$$

where line(x,y,z) is the aggregated summation of the row, and plane(x,y,z) is the aggregated summation of the plane. The initial value of the line(x,−1,z), plane(−1,y,z), and $intVol_{x}\left(x,y,-1\right)$ is zero. Algorithm 1 shows the details of how to use the previous equations to calculate the integral volume in one pass.
**Algorithm 1:** An algorithm to calculate the integral volume in single path
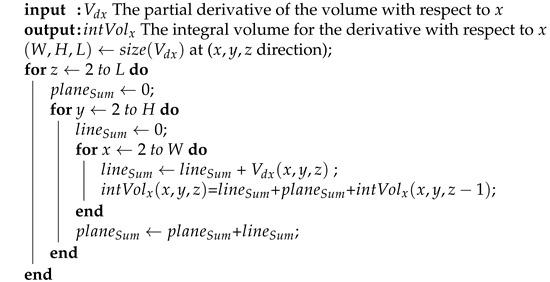


**Figure 5:**
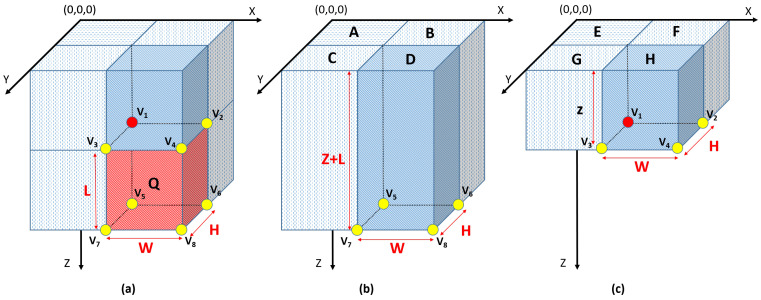
Illustration showing how the integral volume is used in calculating the mean gradient of (**a**) sub-volume *Q*, (**b**) sub-volume *D*, (**c**) sub-volume *H*.

After calculating $\bar{intVol}$, the mean gradient $\bar{N}$ of any 3D sub-volume *Q* is determined by its base point $V_{1}$ and its width (W), height (H), and length (L), as shown in Figure 5a, using the following equations: $\bar{N}=\bar{D}-\bar{H}$; $\bar{D}=\bar{V_{8}}-\bar{V_{6}}-\bar{V_{7}}+\bar{V_{5}}$; and $\bar{H}=\bar{V_{4}}-\bar{V_{2}}-\bar{V_{3}}+\bar{V_{1}}$.

##### Direction Voting

After computing the mean gradient, each voxel in the cell makes a weighted vote, depending on its magnitude, to the direction bins. Each bin direction is determined by the direction of a vector from a platonic solid center to each of its vertices. Utilizing these directions will result in all the bins being equally divided in the 3D volume space. The result of the dot product of the gradient vector and the bin-direction vector is the components of the gradient vector along all the bins’ directions, which need to be normalized as you see in the following equation:

(13) $$C={\left(\begin{array}{c} c_{1},c_{2},\cdots ,c_{n} \end{array}\right)}^{T}=\frac{V.\bar{N}}{∥\bar{N}∥},$$

where:

- *C* is the normalized components vector of the mean gradient vector $\bar{N}$;
- $c_{i}$ is the normalized component of $\bar{N}$ along the axis from the origin to vertex $v_{i}$;
- $\bar{N}$ is the mean gradient vector; and
- *V* is the Cartesian coordinates matrix of the platonic solid vertices.

Table 1 shows the only five platonic solids that can be used for binning, along with their face numbers and vertices coordinates.

Now, the mean gradient components appear in all directions, and they should be thresholded in order to obtain the required components, which should follow the following constrains:

- The vector components appear only on the nearest 3 axes.
- The vector that overlaps any axis must have one component on that axis only.

The threshold value is the value of the component of any axis on any of the adjoining axes. This threshold will be subtracted from all components and the negative components will be set to zero. The final normalized magnitude of the mean gradient–vector components are computed utilizing this equation:

(14) $$\tilde{C}=\frac{∥\bar{N}∥.C}{∥C∥}.$$

Finally all the calculated histograms for the cells are added together on the level of the block and normalized to form the HOG features for all the blocks.

#### 2.1.3. MGRF Energy

The pulmonary nodule volumes are analyzed and modeled using our developed 7th order MGRF model that is used to calculate the Gibbs energy for each voxel in the volume of the nodule [17,36]. This Gibbs energy represents the distinction not only between the voxel HU value, but also between its neighbors. This model will help to avoid the problems of the CT images, such as the acquisition parameters’ variation and the difference between the scanners’ qualities, without affecting the original signal appearance order. The interactions between each voxel and its neighbors are reported as Gibbs energy, and the model is trained on the benign nodules utilizing their approximate maximum likelihood estimates (MLE). The model correlates the relationship between the Gibbs energy with the image texture in a general-case exponential family distribution as follows:

(15) $$P_{z}\left(g\right)=\frac{1}{Z}\;\exp\left(-E_{7}\left(g\right)\right),$$

where g is the HU value in the lung CT scan, $E_{7}\left(g\right)$ is the Gibbs energy, and *Z* is a factor of normalization. The output of this descriptor is the intensity histogram for the Gibbs energy image. This energy will be calculated for each pixel using the above equation. This Gibbs energy image will look brighter for the malignant nodule than the benign one because of its non-homogeneity. See [17] for more details about the higher-order MGRF.

### 2.2. Shape Features

Generally, the pulmonary nodules begin as small spheres. In the case of malignant nodules, this small sphere starts to grow rapidly, which will make it have a very large size in a small period of time and will also make it very difficult for it to maintain its regular shape. This behavior of the malignant nodules makes it have a large and complex shape compared to benign nodules. This variation in the shape between malignant and benign nodules is modeled using three different features, namely, Multi-views Peripheral Sum Curvature Scale Space, spherical harmonics expansion, and some fundamental geometric features, which are described in the following sections.

#### 2.2.1. Multi-Views Peripheral Sum-Curvature Scale Space

**Figure 6:**
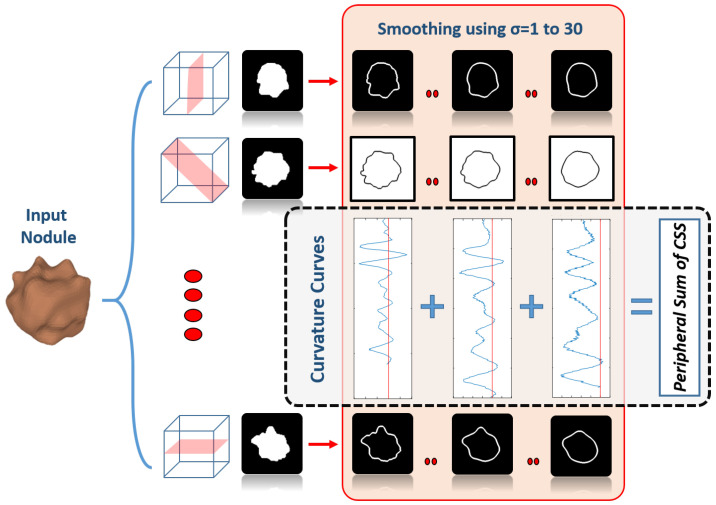
Multi-views Peripheral Sum-Curvature Scale Space illustration that shows the nodule volume and its different 2D projection planes and their edges after detection using Canny algorithm for different Gaussian smoothing parameters.

As mentioned before, the malignant nodule has a sharp contour with multiple spikes. This makes the models that describe the outer shape of the nodule very important for distinguishing between malignant and benign nodules. The multi-views peripheral sum curvature-scale space descriptor is designed to capture the variations in the outer shape by analyzing the changes in the contour curvature nodule circumference. Generally, the curvature at any edge point in a curve can be calculated using the following equation [37]:

(16) $$k\left(t\right)=\frac{\frac{\partial x}{\partial t}\frac{\partial^{2}y}{\partial t^{2}}-\frac{\partial^{2}x}{\partial t^{2}}\frac{\partial y}{\partial t}}{{\left[{\left(\frac{\partial x}{\partial t}\right)}^{2}+{\left(\frac{\partial y}{\partial t}\right)}^{2}\right]}^{\frac{3}{2}}},$$

where $(x\left(t\right)$ and $y\left(t\right))$ are the parametric equations of the edge curve. As the previous equation works on the 2D plane, and the extracted nodules are 3D in volume, multiple 2D views are extracted from the nodule volume from predefined planes. This idea results in the single nodule having multiple 2D images that the descriptor can apply to them. The 2D plans are equally distributed and their number is a parameter to the descriptor ( this number will be optimized in the experimental results). For each 2D image, the Canny algorithm is applied to each section of the nodule to find its edge using different Gaussian scale spaces [38]. The curvature of the edge is calculated at each edge point using Equation (16). The points where the curvature sign is changed are considered to be reversal points, which is an indicator of the nodule contour complexity. As much as the Gaussian smoothing parameter is increased, this reversal points number is decreased until it reaches zero, when the nodule shape looks almost like a single circle. Figure 6 illustrates and summarizes how the multi-views peripheral sum-curvature scale space works. In order to make the descriptor rotation invariant and to make use of the fact that the curvature at any certain point will not change when the nodule is rotated, the number of the reversal point at each Gaussian smoothing parameter is aggregated to form the final feature of each nodule.

#### 2.2.2. Spherical Harmonic

The complexity of the nodule’s surface is an important feature that can be used as a discriminatory feature to differentiate between benign and malignant nodules. This information is intrinsic and based on the fact that benign nodules have smoother or more regular surface/shapes compared to the complexity that exists within the malignant ones. This complexity stems from the hypothesis that the growth rate of malignant nodules is higher than the benign ones and this leads to such irregularity/complexity in their shapes. To capture this characteristic, we used our modeling for the shape complexity description using spherical harmonics’ (SHs) decomposition of the given nodule and extracting the reconstruction error between the original nodule and the constructed one as the input feature to our framework. In the modeling of the shape complexity using SHs, each nodule surface is modeled as a linear combination of basis functions. First, the surface of the nodule is approximated in a discrete way using triangular 3D mesh. This is performed using the TETGEN [39], which is a reliable and fast tool for generating triangular meshes. Consequently, that surface is mapped to the unit sphere using our Attraction–Repulsion algorithm [17] to provide accurate modeling by keeping the distance from the nodule’s center to each node on the surface equal to one and also maintaining an equal distance between each node and all of its neighbors. Following the mapping of the nodule surface to the unit sphere, the nodule is represented as a linear combination of SHs and the order of the harmonics will be used as a representation of the shape complexity. Lower-orders will be enough to represent simple shapes (benign nodules), while higher-orders will be required to represent more complex shapes (malignant nodules). In this work, the solution of the isotropic heat equation for the nodule surface on the unit sphere is used to estimate the coefficients of the linear combination. These coefficients will be used in the reconstruction process using the iterative residual fitting system developed by Shen and Chung [40].

#### 2.2.3. Fundamental Geometric Features

The morphological features of the lung nodules are remarkable characteristics in detecting the nodules’ malignancy. A set of morphological features is calculated for the extracted lung nodule. Table 2 shows the calculated morphological features and their definitions.

To remove the bias stemming from accusation parameters (e.g., pixel spacing and slice thickness), a predetermined volume of interest (VOI) of size 40×40×40 mm${}^{3}$ centered around each nodule’s center is extracted and resampled to be isotropic in all directions.

## 3. Experimental Results

The testing of all features was based on multiple stages of classification. The first stage is responsible for providing a preliminary diagnosis probability of malignancy for the nodule for each descriptor. The second stage is responsible for chaining all the output probabilities together to give the final nodule diagnosis based on the fusion of all the preliminary diagnosis probabilities. The architecture of the first stage for all features consists of three hidden auto-encoder layers. Each of the layers reduces the dimensionality of its input by one third, and the last layer just passes its output to a softmax classifier to give the preliminary diagnosis probability of malignancy for the nodule. The second stage starts with one hidden auto-encoder that feed its output into a softmax classifier to give the final nodule diagnosis.

To validate our proposed system, we utilized two datasets. The first dataset is the publicly accessible Lung Image Database Consortium and Image Database Resource Initiative (LIDC/IDRI) dataset [41]. We chose it, as it is the most significant public lung cancer diagnosis benchmark dataset, allowing us to contrast our solution against competing frameworks. The dataset includes 1080 thoracic CT images from 1010 individuals gathered from 8 medical imaging companies and 7 academic institutes. It is linked with annotation files in eXtensible Markup Language (XML) format. The labeling process is performed by four radiologists through two phases, and the results are recorded in the XML files. The first phase is a blinded phase, in which each radiologist categorizes the lesions into one of three groups: nodule ≥3 mm, nodule <3 mm, or non-nodule ≥3 mm. The second phase is an unblinded phase in which radiologists interact to give their final assessment. The final assessment includes nodule boundaries, as well as subjective nodule feature evaluations such as solidity, spiculation, margin, internal structure, shape (sphericity), lobulation, subtlety, and probability of malignancy. The grading for all lesion features is specified as a discrete categorical field with the values 1, 2, 3, 4, or 5, with 1 representing the weakest value of the feature and 5 representing the strongest value of the feature. At least one radiologist labeled 7371 lesions as a nodule in the dataset. Moreover, at least one radiologist designated 2669 of these lesions as nodule ≥3 mm with 928(34.7%) receiving such labels from all four radiologists. Since the malignancy grade is dependent on four radiologists’ opinions, which may conflict, and because this malignancy is not based on biopsy, we formed our training and testing datasets by choosing a nodule ≥3 mm with an appropriate degree of agreement between the four radiologists. The average grading of the four radiologists was used to obtain the final grading for each nodule, where a grading ≥3.5 was considered malignant, while a grading ≤1.5 was considered benign. These standards resulted in a validation dataset of 727 nodules from 467 individuals. This validation dataset is almost balanced, as it includes 314 malignant nodules (43.2%) and 413 benign ones (56.8%).

The second dataset is our locally acquired CT imaging dataset that was collected from 80 patients during the period from 2016 to 2018 by our medical collaborators at the University of Louisville Hospital. The Institutional Review Board (IRB) at the University of Louisville approved the research protocol, and all the procedures were conducted with respect to the relevant guidelines and regulations. The scans were collected with a slice thickness of 2.5 mm, tube voltage of 140 KV, tube amperage of 100 mA, and a reconstruction interval every 1.5 mm. Each CT scan was annotated by three experienced radiologists, the final contour of the nodule was the union of their manual segmentation, and there was no notable difference between their diagnoses.

To accommodate for the differences in pixel resolution and slice thickness between the CT scanners in the two datasets we used, we retrieved all nodules using a 40 mm cube window around the nodule centroid. In order to evaluate our framework, the data was split into training (70%) and testing (30%). The system performance was measured using different performance metrics, namely, accuracy (AC), sensitivity (SN), specificity (SP), precision (PR), and the area under the receiver-operating characteristics curve (AUC). There were a lot of parameters that needed to be optimized for each feature descriptor. For the HOG feature there was a need to optimize the following parameters:

- The number of cells in each block for which the mean gradient was to be calculated.
- The number of blocks that the volume was divided into.
- The number of histogram bins.
- The binning style, either full binning or half.

Table 3 presents different evaluation metrics for the HOG features using 20 full bins. It shows that using 5×5×5 blocks and 3×3×3 cells were optimal for this experiment. Table 4 presents the same evaluation metrics using 5×5×5 blocks and 3×3×3 cells for the different binning options. It shows that using a dodecahedron (20 bins) and full binning are the optimum parameters for this experiment.

Multi-view ALBP has many parameters that require tuning in order to achieve the best accuracy. These parameters are:

- The number of levels used around the center point.
- The scheme used for resampling.
- The number of extracted 2D-views (the 2D-views are equally distributed).

Table 5, Table 6 and Table 7 present different evaluation metrics for the Multi-view analytical LBP features using full N resampling, single resampling, and average N resampling schemes, respectively. It shows that using 5 views and 3 levels is optimal for these experiments.

The output of the MGRF is shown in the Gibbs energy image for the nodule. This image has a high Gibbs energy for benign nodules and a low energy for malignant ones. The histogram for this image is selected as a representation for the Gibbs energy image. The high-energy bins for the malignant nodules have lower densities compared to the low-energy bins that have high densities. The number of histogram bins is the parameter that needs to be optimized. Table 8 shows the reported evaluation metrics for different bin numbers. It can be concluded from this table that there was no noticeable difference in the evaluation metrics when the number of bins changed. This stability in the evaluation metrics, regardless of the change of bin numbers, makes the MGRF and Gibbs energy a reliable feature for distinguishing between the appearance of the malignant and benign nodules.

The Multi-View PSCSS descriptor parameters that need to be tuned are:

- The pixel gap while calculating the curvature.
- The number of extracted 2D views.

Table 9 presents different evaluation metrics for the Multi-view PSCSS features while using different values for the number of extracted views and the pixel gap while calculating the curvature. The perfect combination is a gap of 15 pixel and 5 2D views.

**Figure 7:**
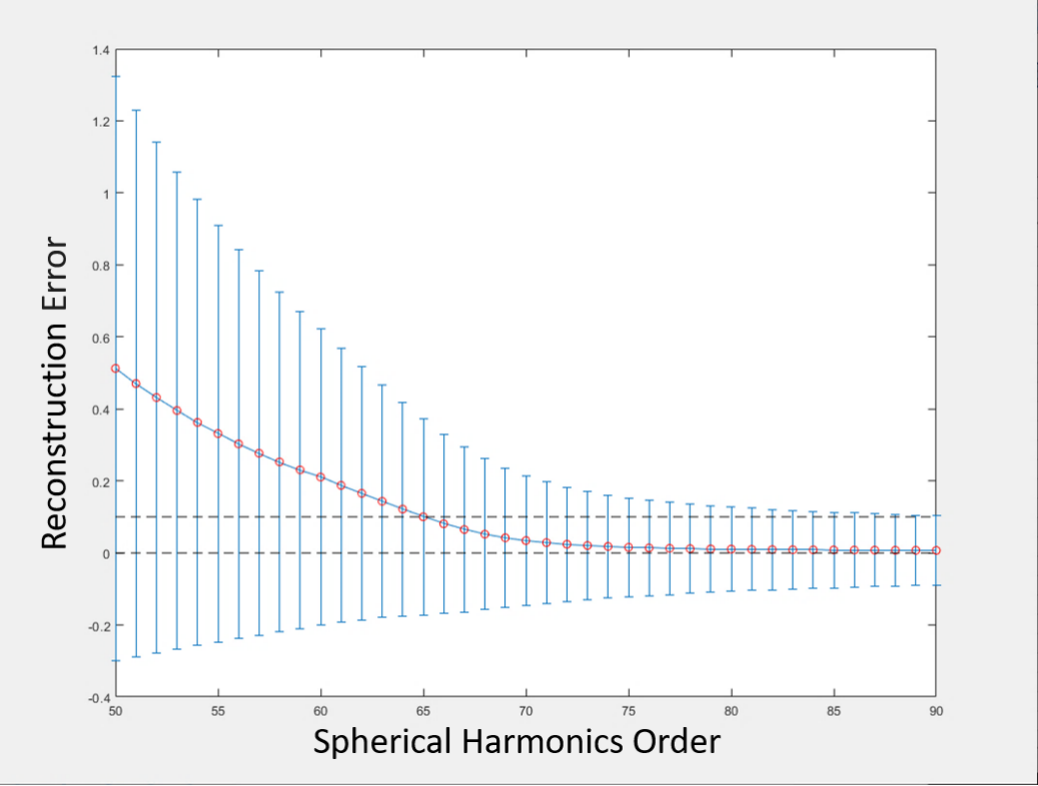
Average error curves of the nodule reconstruction using different spherical harmonics orders.

The spherical harmonic expansion can use different orders of spherical harmonics to reconstruct the lung nodule. Using higher orders will reduce the reconstruction error compared to the low orders. The order number of the spherical harmonics is the parameter that needs to be optimized to reduce the redundant calculations while building the feature vector. Figure 7 shows the average error curve for the reconstruction of all the nodules. It can be concluded from the figure that the average reconstruction error is less than 0.1 after 65 spherical harmonics orders. Although the 65 orders are enough to describe the used data, the feature vector that will be used in the classification will be 70 in order to avoid over-fitting the utilized data.

Table 10 shows the detailed performance measures for each descriptor individually and for the system as a whole. Table 11 shows the performance comparison between the proposed features and the whole system with some recent frameworks using the LIDC dataset only. All the system components were developed on Matlab R2019B.

## 4. Discussion and Conclusions

The experimental results demonstrate that the presented non-invasive CAD system for the lung nodule diagnosis is promising and has achieved very high accuracy measures from a single computed tomography scan. Currently, the classification of the pulmonary nodule, whether malignant or benign, depends on measuring the growth rate of the nodule from multiple CT scans by following it up for approximately two years to avoid performing a biopsy, which is an invasive procedure for diagnosis. The repeated CT scan cost is very high, and it also exposes the patient to a lot of radiation that may affect their health badly. The key point behind increasing the accuracy measures of our proposed CAD system is combining both texture-appearance features and shape features. The pulmonary nodule starts as a small sphere, and, with time, it gets larger. In the case of the malignant nodules, their rapid growth makes it very difficult to maintain their smooth contour, which results in the irregular shape of the nodules. The implemented shape descriptors are able to capture this irregularity in the nodules’ outer shape and provides a good prediction for the prior growth rate of the nodule, which eliminates the need for multiple scans. In the same way, the nodules’ texture appearance starts smooth and homogeneous, but, due to the malignant nodule’s high growth rate, it starts to have a non-homogeneous texture, and the implemented appearance descriptors are able to capture this non-homogeneity in the nodule texture to give another prediction for the prior nodule growth rate. One of the interesting points, when we compare the accuracy measures of the proposed system with the one proposed in [17], is that the increase in the accuracy is small (from 93.97% to 94.73%). This small increase is predictable, as all the features used by both systems model the same nodule characteristics (both shape and appearance). However, the system proposed in this manuscript increased the sensitivity of the system from 90.48% to 93.97%, which is a significant increase and shows how the new system with the added features is less sensitive to the segmentation procedure and the image artifacts.

Currently, more than 65% of lung cancer patients are detected in a late stage, which makes it very difficult for them to recover. Thus, there is a real need for a non-invasive CAD system that reduces the diagnosis time from multiple scans to a single CT scan. The proposed framework is robust and can be extended to use new markers, as it depends on integrating the final results of each component separately. This manuscript presents a non-invasive CAD system for the detection of the lung cancer from a single computed tomography scan. It uses two groups of features, which are appearance (texture) features and shape (contour) features. The experimental results show the ability of theses descriptors to precisely differentiate between malignant and benign nodules. The main advantage of the presented system is its ability to classify the pulmonary nodules from a single computed tomography scan instead of repetitive scans. The extracted features were able to predict a nodule’s prior growth rate by examining the nodule’s texture and shape, which is the main point for pulmonary nodule diagnosis.

## Figures and Tables

**Table 1:** Different platonic solids and their faces and vertices.

Platonic Solid	Tetrahedron	Octahedron	Cube	Icosahedron	Dodecahedron
Vertex Coordinates	(1,1,1) (1,−1,−1) (−1,1,−1) (−1,−1,1)	(±1,0,0) (0,±1,0) (0,0,±1)	(±1,±1,±1)	(0,±1,±ϕ) (±1,±ϕ,0) (±ϕ,0,±1)	(±1,±1,±1) (0,±1/ϕ,±ϕ) (±1/ϕ,±ϕ,0) (±ϕ,0,±1/ϕ)

Note: ϕ is the golden ration that equal $\frac{1+\sqrt{5}}{2}$.

**Table 2:** Example of some extracted geometric features.

Feature	Details
Volume	The actual count of the voxels inside the nodule’s region of interest.
Convex Volume	The count of voxels inside the nodule’s convex region.
Equivalent Diameter	The diameter of the equivalent sphere that has the same volume as the nodule.
Surface Area	The area of the boundaries of the nodule.
Solidity	The ratio between the number of voxels in the nodule and the number of voxels in the nodule convex.
Principal Axis Length	The length of the major axes of the ellipsoid that have the same normalized second central moments as the nodule volume.
Extent	The ratio between the number of voxels in the nodule and the number of voxels in the nodule-bounding box.

**Table 3:** Comparison between accuracy, sensitivity, and specificity using different configurations of 3D-HOG parameters (number of blocks and cell size).

Number of Blocks	Cell Size	AC	SN	SP
4	3	83.03	80.65	84.80
	4	79.36	72.04	84.80
	5	81.65	74.19	87.20
	8	71.56	60.22	80.00
	10	78.90	68.82	86.40
5	3	88.17	87.26	88.86
	4	86.24	82.80	88.80
	5	83.03	78.49	86.40
	8	84.86	81.72	87.20
	10	80.28	82.80	78.40

**Table 4:** Comparison between accuracy, sensitivity, and specificity using different configurations of 3D-HOG parameters (the polyhedron type and binning style).

Polyhedron	Binning Style	AC	SN	SP
Dodecahedron (20 bin)	Full binning	88.17	87.26	88.86
	Half binning	70.80	74.49	68.00
Icosahedron (12 bin)	Full binning	72.61	85.25	63.20
	Half binning	66.56	55.22	75.00

**Table 5:** Comparison between accuracy, sensitivity, and specificity for full N sampling using different configurations of Multi-View Analytical LBP parameters (number of views and number of levels).

		Full N Resampling		
**Views Number**	**Levels Number**	**AC**	**SN**	**SP**
3	2	85.13	82.45	88.32
	3	88.53	87.62	88.68
	4	86.32	76.12	91.23
	5	84.86	80.65	85.10
5	2	88.91	87.31	89.37
	3	90.69	91.50	89.60
	4	90.56	91.32	89.40
	5	89.78	87.10	90.65
7	2	87.75	78.33	90.30
	3	87.90	80.17	90.21
	4	86.24	79.11	89.70
	5	85.32	77.30	86.67

**Table 6:** Comparison between accuracy, sensitivity, and specificity for single N sampling using different configurations of Multi-View Analytical LBP parameters (number of views and number of levels).

		Single N Resampling		
**Views Number**	**Levels Number**	**AC**	**SN**	**SP**
3	2	83.13	80.56	84.70
	3	88.17	87.30	88.56
	4	84.33	81.72	87.30
	5	83.80	81.72	86.40
5	2	86.50	82.80	88.71
	3	88.71	87.60	88.93
	4	87.10	79.85	89.31
	5	84.78	81.33	85.11
7	2	86.50	76.20	90.00
	3	87.10	79.85	88.93
	4	85.95	78.91	87.56
	5	83.80	81.72	86.40

**Table 7:** Comparison between accuracy, sensitivity, and specificity for average N sampling using different configurations of Multi-View Analytical LBP parameters (number of views and number of levels).

		Average N Resampling		
**Views Number**	**Levels Number**	**AC**	**SN**	**SP**
3	2	84.31	82.86	86.40
	3	88.80	87.51	89.13
	4	85.60	80.36	87.66
	5	86.00	76.73	90.65
5	2	88.81	87.33	89.70
	3	90.73	92.56	90.31
	4	90.18	90.80	89.63
	5	89.90	91.32	88.13
7	2	88.00	87.13	89.70
	3	89.60	88.12	90.57
	4	88.20	87.10	89.73
	5	87.80	80.45	88.44

**Table 8:** Comparison between accuracy, sensitivity, and specificity using different histogram bin numbers for the Gibbs energy image.

Bins Number	Accuracy	Sensitivity	Specificity
400	89.32	92.37	87.94
600	89.83	87.50	92.75
800	89.12	92.11	88.59
1000	89.91	93.55	87.20

**Table 9:** Comparison between accuracy, sensitivity, and specificity using different configurations of Multi-view PSCSS parameters (number of views and gaps between points).

Views Number	Points Gap	AC	SN	SP
3	10	87.61	87.10	88.00
	15	88.53	88.17	88.80
	20	85.32	84.95	85.60
	25	84.86	82.80	86.40
5	10	89.91	89.25	90.40
	15	90.37	89.25	91.20
	20	85.78	87.10	84.80
	25	84.40	83.87	84.80
7	10	88.53	87.10	89.60
	15	87.61	87.10	88.00
	20	86.24	84.95	87.20
	25	85.32	82.80	87.20

**Table 10:** Evaluation of each feature and the whole framework using LIDC and our locally acquired dataset.

	Evaluation Metrics				
	Accuracy	Sensitivity	Specificity	Precision	AUC
3D-HOG	87.54	86.65	87.53	84.37	0.941
Multi-view Analytical LBP	90.15	89.70	91.88	89.37	0.9535
MGRF	90.23	93.87	86.75	84.62	0.9533
Multi-view PSCSS	89.53	89.39	90.65	87.11	0.9534
Spherical Harmonics	88.60	92.32	86.88	84.58	0.9593
Geometric features	82.11	77.46	85.33	84.01	0.8903
Proposed System	92.55	91.70	93.40	90.93	0.9616

**Table 11:** Comparison between the proposed features and the whole system with some recent frameworks using LIDC dataset only.

	Evaluation Metrics				
	Accuracy	Sensitivity	Specificity	Precision	AUC
3D-HOG	88.17	87.26	88.86	85.62	0.9462
Multi-view Analytical LBP	90.73	90.57	92.19	89.87	0.9587
MGRF	89.91	93.55	87.20	84.47	0.9666
Multi-view PSCSS	90.37	89.25	91.20	88.61	0.9666
Spherical Harmonics	89.82	93.55	87.20	84.75	0.9642
Geometric features	80.73	76.64	84.68	82.83	0.8720
Proposed System	94.73	93.97	95.13	93.56	0.9867
Gupta et al. [42]	81.50	78.11	85.64	-	-
Safta et al. [43]	93.10	91.11	95.24	95.35	0.9767
Shen et al. [44]	84.20	70.50	88.90	67.25	0.8560
Ren et al. [45]	90.00	81.00	95.00	-	-
Wei et al. [46]	87.65	89.30	86.00	86.45	0.942
Sang et al. [47]	92.00	94.00	90.00	-	0.99

## Data Availability

Part of the data presented in this study are publicly available in the Lung Image Database Consortium image collection (LIDC-IDRI) at https://wiki.cancerimagingarchive.net/display/Public/LIDC-IDRI (accessed on 26 January 2022). Our locally acquired datasets presented in this study are not publicly available as they are protected under IRB number (10.0642).
